# Male partner attendance of skilled antenatal care in peri-urban Gulu district, Northern Uganda

**DOI:** 10.1186/1471-2393-10-53

**Published:** 2010-09-16

**Authors:** Raymond Tweheyo, Joseph Konde-Lule, Nazarius M Tumwesigye, Juliet N Sekandi

**Affiliations:** 1 Makerere University School of Public Health, Department of Health Policy Planning, and Management. P.O. Box, 7072, Kampala, Uganda; 2 Makerere University School of Public Health, Department of Epidemiology and, Biostatistics, P.O.Box, 7072, Kampala, Uganda

## Abstract

**Background:**

Male partner attendance of skilled Antenatal Care (ANC) is beneficial to improving maternal outcomes. This study investigated the level, perceived benefits and factors associated with male partner attendance of skilled ANC in a peri-urban community recovering from two decades of civil conflict.

**Methods:**

This cross-sectional survey used multi-stage sampling in 12 villages of Omoro county to select 331 married male respondents aged 18 years or more, whose female spouses had childbirth within 24 months prior to the survey. A structured questionnaire elicited responses about male partner attendance of ANC during pregnancy at a public health facility as the main outcome variable. Analysis used Generalized Linear Model (GLM) in Stata version 10.0 to obtain Prevalence Risk Ratios (PRR) for association between the binary outcome and independent factors. All factors significant at p < 0.15 and potential confounders were included in the multivariable model.

**Results:**

Overall, 65.4% (95%CI; 60.3, 70.5) male partners attended at least one skilled ANC visit. Mean age was 31.9 years [SD 8.2]. Perceived benefits of attending ANC were: HIV screening (74.5%), monitoring foetal growth (34%) and identifying complications during pregnancy (18.9%). Factors independently associated with higher ANC attendance were: knowledge of 3 or more ANC services (adj.PRR 2.77; 95%CI 2.24, 3.42), obtaining health information from facility health workers (adj.PRR 1.14; 95%CI 1.01, 1.29) and if spouse had skilled attendance at last childbirth (adj.PRR 1.31; 95%CI 1.04-1.64). However, factors for low attendance were: male partners intending their spouse to carry another pregnancy (adj.PRR 0.83; 95%CI 0.71, 0.97) and living more than 5 Km from a health facility (adj.PRR 0.83, 95%CI 0.70, 0.98).

**Conclusions:**

Men who were knowledgeable of ANC services, obtained health information from a health worker and whose spouses utilised skilled delivery at last pregnancy were more likely to accompany their spouses at ANC, unlike those who wanted to have more children and lived more than 5 km from the health facility. These findings suggest that empowering male partners with knowledge about ANC services may increase their ANC participation and in turn increase skilled delivery. This strategy may improve maternal health care in post conflict and resource-limited settings.

## Background

Worldwide, male attendance of skilled ANC and delivery care remains a challenge to safe motherhood. About 210 million women become pregnant each year with 30 million (15%) developing complications, resulting into over half a million maternal deaths [1]. Developing countries account for more than 99% of all maternal deaths; about a half occurring in sub-Saharan Africa, and a third in South Asia [2,3]. There is slow progress towards achieving the fifth Millennium Development Goal (MDG) in developing countries[4]. Uganda continues to have one of the highest maternal and child mortality worldwide, with an estimated Maternal Mortality Ratio (MMR) at 435/100,000 and child mortality at 137/1,000 live births [5]. The Ugandan government has prioritized reproductive health strategies which focus on accelerated reduction in maternal mortality and severe morbidity related to pregnancy and childbirth.

As early as 2001, the World Health Organization (WHO) established proven safe motherhood interventions that are required at household, community and facility levels to enable every pregnant woman to have a safe pregnancy and childbirth, and to provide couples with the best chance of having healthy infants [6]. The strategies include; providing skilled attendants to prevent, detect and manage the major obstetric complications, together with providing equipment, drugs and other supplies[7,8]. A few studies have found that facility interventions providing routine ANC have minimal beneficial effects to maternal health outcomes, creating a rationale for community-based interventions for pregnant women where educational and support programmes have been successful in increasing/improving skilled delivery, breastfeeding practices and postnatal attendance [9-12]. This reported success may be mainly attributable to spousal involvement, and not necessarily the community as a whole. Even though it is widely recognized that there is limited research on the role of male involvement during pregnancy [13-15], there are promising links to beneficial effects. Indeed some few prospective studies and literature reviews have successfully demonstrated that prenatal male involvement is associated with beneficial health outcomes such as; higher first trimester ANC visits, abstinence from smoking and alcohol consumption [14,16], and reduction in low birth-weight infants [14,17]. Evaluations of Prevention of Mother-to-Child Transmission of HIV (PMTCT) which is often implemented within the confines of safe motherhood have similarly shown male involvement to positively influence uptake of HIV testing and preventive interventions for vertical and sexual transmissions of HIV [15,18,19].

Male attendance of skilled ANC is a fairly new field to research in Uganda. The available estimates depict a low attendance averaging 3% in 2006 [20] but are based on health facility information systems that monitor male attendance in the PMTCT programme. Gulu district is located in northern Uganda, a region recovering from a 20-year-old insurgency. The civil war forced majority of its population into Internally Displaced Peoples' (IDP) camps. According to Gulu development plan (2007), the district has a maternal mortality ratio (MMR) of 700/100,000; infant mortality rate of 172/1,000 and ANC HIV sero-prevalence of 8.8% [21]. Gulu district MMR is similar to other districts in Northern Uganda with an MMR averaging 650/100,000 but, higher than neighbouring post-conflict affected Adjumani district in West Nile where the MMR is estimated at 550/100,000 [21]. In 2008, the district estimate for male attendance of skilled ANC was 18.4% having risen from about 3% in 2006 [22]. Of the total 23,408 pregnant women that were new ANC attendees in Gulu district in 2008, male attendance estimated at 18.4% varied by sub-county; 41.6% (1,107/2,746) in Aswa, 41.5% (2,529/6,264) in Omoro and only 5.0% (660/14,398) in the Municipality[22]. The marked rise in male partner ANC attendance is possibly due to a robust district-wide PMTCT programme. This study assessed the level, perceived benefits and factors associated with male partner attendance of skilled ANC in a peri-urban community in Gulu.

## Methods

### Study setting

Gulu district is located in northern Uganda, with a population of 343,100 people [21] and approximately 17,155 (5%) women expected to become pregnant annually. The annual population growth rate is 3.3% with a sex ratio of 95 males to 100 females [5]. The district health structure is made up of 48 health facilities, with about 77% (32) functional, most of which provide antenatal care services.

### Study design, participants and sampling procedure

A cross-sectional survey design was used employing quantitative techniques. Study participants were male partners to women of reproductive age (18 - 49 years) that had a full-term delivery within the last 24 months preceding the study in Gulu district. Sample size was estimated using the modified Kish Leslie (1965) survey sampling formula with a 95% confidence interval, a precision of 5%, an estimated proportion of male partners attending ANC of 18.4% and a conservative design effect of 1.5 to cater for intra-cluster variability[23]. The calculated sample size was 346 male respondents.

Omoro, one of the three counties in Gulu district was selected by simple random sampling. All parishes in the six sub-counties of Omoro (Odek, Lalogi, Lakwana, Bobi, Koro and Ongako) were listed. We selected 12 out of the total 28 parishes in all the sub-counties by simple random sampling. Consecutive recruitment of 29 participants in each cluster at parish level was done. The first participant was obtained from an eligible household along the primary road of the index cluster, which was located about 200 metres from the commercial focal point. The subsequent respondents were recruited by carrying out a door-to-door assessment of eligibility, along the primary, secondary and tertiary roads, up to 3 kms away from the commercial focal point of the cluster. In the absence of an eligible respondent, the subsequent households were screened until a suitable respondent was found. In case of an eligible polygamous male partner, the information was asked about the wife that last delivered, irrespective of her rank. The process of identifying eligible respondents was continued until 29 participants were found in each cluster. On average, an eligible respondent was found from one in six households visited. Data were collected through face to face interviews using a pre-tested semi-structured questionnaire.

### Data management and analysis

The main outcome variable was male partner attendance of at least one skilled ANC visit of the spouse's last full-term pregnancy. Double data entry and validation was done in Epi data 3.1. Data was then exported to Stata 10.0 where frequencies and statistical analyzes were run. The outcome variable of the study [male attendance of ANC] was a binary categorical variable (attended, not attended). All independent variables had nearly same sample size as the outcome variable (n = 331) with the highest item non response at 4.8% (16/331). Since the prevalence of the outcome was found to be high at (65.4%), odds ratios were not a good approximation of the measure of association[24,25]. We computed Prevalence Risk Ratios (PRR) for association between the binary outcome [male partner attendance of at least one ANC visit, versus non-attendance], in relation to independent variables such as age and education level using GLM analysis with binomial family and a log link with robust standard errors [24,25]. All factors significant at p < 0.15 or potential confounders and socially plausible factors were included in the multivariable model to obtain adjusted PRR with their 95% confidence intervals (95%CI). Analysis was conducted using Stata version 10.0. We then did stepwise backward elimination until the best fitting model with a log likelihood tending toward zero was obtained. We identified statistically significant adjusted PRR from the model and reported them as independent factors associated with male attendance of skilled ANC.

### Ethical considerations

The study obtained ethical approval from Makerere University School of Public Health (MUSPH) Higher Degrees and Ethical Committee. Written informed consent was obtained from adult respondents aged 18 years and above, clearly stating potential risks and benefits of the study and sought their voluntary participation.

## Results

### Characteristics of study sample

Table 1 shows the characteristics of male partners included in the study. A total of 331 adult male partners aged ≥18 years were interviewed representing a response rate of 95.5%. Nearly all respondents were household heads 99.1% (328/331), with a mean (SD) age of 31.9 (SD = 8.2) years; range 18 - 62 years. Out of the 331 respondents, majority were peasant farmers (81.5%), most lived in rural areas (61.3%), nearly all (97.9%) were co-resident (staying together) with their female spouses. About 60.7% were religiously or traditionally married, and 17% were in polygamous marital unions.

**Table 1:** Characteristics of male respondents

Variable	Freq N = 331	Proportion (%)
**Age**^a^		
15-24	63	19
25-34	149	45
35-44	88	26.6
≥45 years	31	9.4
**School education**		
None	12	3.7
Primary	209	64.7
Secondary	78	24.1
Tertiary	23	7.1
Missing data	8	__
**Residence**		
Semi-urban	201	61.3
Urban	128	38.7
Missing data	3	__
**Household size**^b^		
≤ 4	112	33.9
> 4	218	66.1
Missing data	1	___
**Marital Status**		
Never married	112	33.8
Married	201	60.7
Living together	17	5.1
Missing data	1	__
**Type of marriage**		
Monogamous	273	83
Polygamous	56	17
Missing data	2	__
**Religion**		
Protestant	69	21
Catholic	222	67.7
Other	37	11.3
Missing data	3	__
**Occupation**		
Peasant farmer	268	81.5
Manual	20	6.1
Sales	19	5.8
Professional	22	6.7
Missing data	3	__
**No. of children**^c^		
≤ 3	168	51.7
> 3	157	48.3
Missing data	6	__

^a^Mean age 31.9, ^b^Median household size = 5 (IQR 4 - 8), ^c^Median no. of children = 3 (IQR 2 - 5).

### Level of male attendance of ANC, knowledge and perceptions

Respondents were asked whether or not they attended ANC with their spouses during their last full-term pregnancy, what they knew about ANC services offered, their willingness to attend ANC in subsequent pregnancies, and what they perceived as benefits and barriers of attendance. The proportion of male partners that participated in at least one ANC visit was 65.4% (95%CI 60.3, 70.5). Their willingness to attend subsequent ANC visits with their spouses was also high, 93.7% (95%CI 91.1, 96.3). Most men attended no more than two ANC visits (60.5%), but this attendance was more common at government health facilities compared to other facilities, (82.5% vs 17.5%, p < 0.001). The ANC visits were mostly begun during the spouse's first trimester (59.8%).

About 96% (318 out of 331) respondents regarded attendance of spousal ANC as beneficial. The major perceived benefits of attending ANC were HIV screening (74.5%), monitoring foetal growth (34%), and identifying complications in pregnancy (18.9%). Male partners' knowledge about ANC services offered was limited; about half, 49.9% (164/329) could correctly mention two or fewer services offered, 47.1% (155/329) mentioned 3 - 5 services, and only 3% (10/329) mentioned at least five services offered.

The main barriers to attendance of ANC identified by male partners were: long waiting time (41.7%), lack of transport means (35.8%), walking distance greater than one hour to a health facility (34.1%) and fear of being tested for HIV (29.7%). Other barriers reported include; non-invitation, attaching no importance to their attendance and having a concurrent task or job demand at the same time as the spouse's ANC visit.

### Factors associated with male attendance of ANC

Table 2 shows bivariate analysis of factors associated with male attendance of skilled ANC. The attendance rate was about 3 times higher among male partners that could identify at least three services offered at ANC compared to those identifying two or less services, (PRR 2.72; 95%CI 2.20, 3.36; p < 0.001). Also, the attendance rate was significantly higher if the spouse's last delivery took place in a health facility compared to those where the last delivery occurred either at home or at a traditional birth attendant's place, (PRR 1.42; 95%CI 1.08, 1.88; p = 0.012), or if the male was religiously/traditionally married compared to consensual marital unions (PRR 1.26; 95%CI 1.04, 1.52; p = 0.014). Other factors associated with higher male attendance were attainment of secondary or higher level education (PRR 1.25; 95%CI 1.08 - 1.46; p = 0.003), and perception of foetal monitoring as being a benefit of attendance (PRR 1.20; 95%CI 1.03, 1.40; p = 0.018).

**Table 2:** Bivariable analysis of independent variables associated with male attendance of skilled ANC

Independent Variable			Unadjusted		
	Attended Freq, N = 331(%)	Total	PRR	(95% CI)	p-value
**Age**					
15 - 24	41(65.0)	63	1		
25 - 34	94(63.9)	147	0.98	(1.78 - 1.22)	0.875
35 - 44	58(65.9)	88	1.01	(0.80 - 1.28)	0.916
≥ 45	22(70.9)	31	1.09	(0.81 - 1.45)	0.557
**School education**					
≤ Primary	133(62.1)	214	1		
≥Secondary	78 (76.4)	102	1.25	(1.08 - 1.46)	0.003**
**Residence**					
Semi-urban	131(65.5)	200	1		
Urban	83(65.8)	126	1.00	(0.86 - 1.18)	0.945
**Household size**					
≤ 4	72 (64.2)	112	1		
> 4	142 (65.7)	216	1.02	(0.86 - 1.21)	0.795
**Marital status**					
Never married	63(56.2)	112	1		
Married	142(71.0)	200	1.26	(1.04 - 1.52)	0.014*
Not married	10(58.8)	17	1.04	(0.67 - 1.60)	0.839
**Type of marriage**					
Monogamous	182 (66.9)	272	1		
Polygamous	33 (60.0)	55	0.89	(0.71 - 1.13)	0.356
**Occupation**					
Manual worker	185(64.6)	286	1		
Sales	15 (78.9)	19	1.22	(0.95 - 1.56)	0.115
Professional^a^	14 (63.6)	22	0.98	(0.70 - 1.36)	0.922
**Reported distance to nearest health facility**					
≤ 5 KM	178 (67.7)	263	1		
> 5 KM	37 (56.0)	66	0.82	(0.65 - 1.04)	0.108
**Main sources of health information**					
Radio					
No	47(22.1)	75	1		
Yes	166(77.9)	252	1.05	(0.86 - 1.27)	0.618
VHT member					
No	136(63.8)	218	1		
Yes	77(36.2)	109	1.13	(0.96 - 1.32)	0.126
Health worker at facility					
No	115(54.0)	189	1		
Yes	98(46.0)	138	1.16	(0.99 - 1.36)	0.053
Community campaign					
No	183(85.9)	263	1		
Yes	30(14.1)	64	0.67	(0.51 - 0.88)	0.005**
**Knowledge about services offered at ANC**					
≤ 2 services	57(35.2)	162	1		
3-5 services	158(95.8)	165	2.72	(2.20 - 3.36)	0.000***
**Duration since spouse last carried complete pregnancy**					
≤ 1 year	38 (80.9)	47	1		
**>**1 year	177 (62.8)	282	0.77	(0.65 - 0.91)	0.003**
**Last delivery of spouse was at health facility**					
No	28 (48.3)	58	1		
Yes	187 (69.0)	271	1.42	(1.08 - 1.88)	0.012*
**Intends spouse to carry another pregnancy**					
No	35 (76.1)	46	1		
Yes	177 (63.7)	276	0.83	(0.69 - 1.00)	0.059
**Would attend ANC in subsequent pregnancy**					
No	09(4.3)	20	1		
Yes	199(95.7)	295	1.43	(0.99 - 2.06)	0.054
**Monitoring Feotal growth as perceived benefit**					
No	131(62.4)	209	1		
Yes	79(37.6)	107	1.20	(1.03-1.40)	0.018*
**Walking distance > 1 hour to health facility as perceived barrier**					
No	145(70.4)	199	1		
Yes	61(29.6)	102	0.88	(0.73 - 1.05)	0.176

*Statistically significant at p < 0.05, **p < 0.01, ***p < 0.001 ^a^Professional occupation includes teacher, health worker and engineer

On the other hand, attendance was significantly lower if the source of health information were community campaigns (PRR 0.67; 95%CI 0.51, 0.88; p = 0.005) or if female spouse's last full term pregnancy was more than one year ago (PRR 0.77; 95%CI 0.65, 0.91; p = 0.003).

### Multivariable analysis

After adjusting for confounding in a multivariable analysis, shown in Table 3, the factors that remained significantly associated with male attendance of ANC were: knowledge of at least 3 services offered at ANC (adj.PRR 2.77; 95%CI 2.24, 3.42; p < 0.001), last delivery was at a health facility (adj.PRR 1.31; 95%CI 1.04, 1.64; p = 0.020) and having a health facility worker as the source of health information (adj.PRR 1.14; 95%CI 1.01, 1.29; p = 0.023). Conversely, factors significantly associated with lower attendance rate were: long distance to nearest health facility (at least 5 KM) or longer than one hour's walk (adj.PRR 0.83, 95%CI 0.70, 0.98; p = 0.034), or if male intended female spouse to carry another pregnancy (adj.PRR 0.83; 95%CI 0.71, 0.97; p = 0.023).

**Table 3:** Multivariable analysis of factors associated with male attendance of skilled ANC

OUTCOME: Male Attendance of skilled ANC				
	**Attended**			
	Freq, N = 331(%)			
**Independent Variable**			**Unadjusted (95%CI)**	**Adjusted (95%CI)**
			**PRR**	**PRR**

**Age**				
15 - 24	41(65.0)	63	1	1
25 - 34	94(63.9)	147	0.98 (1.78 - 1.22)	0.91 (0.75 - 1.11)
35 - 44	58(65.9)	88	1.01 (0.80 - 1.28)	0.93 (0.75 - 1.17)
≥ 45	22(70.9)	31	1.09 (0.81 - 1.45)	0.84 (0.66 - 1.08)
**Marital status**				
Never married	63(56.2)	112	1	1
Married	142(71.0)	200	1.26 (1.04 - 1.52)*	1.11 (0.95 - 1.30)
Not married	10(58.8)	17	1.04 (0.67 - 1.60)	0.98 (0.75 - 1.30)
**Type of marriage**				
Monogamous	182 (66.9)	272	1	1
Polygamous	33 (60.0)	55	0.89 (0.71 - 1.13)	0.86 (0.74 - 1.00)
**Reported distance to nearest health facility**				
≤ 5 KM	178 (67.7)	263	1	1
> 5 KM	37 (56.0)	66	0.82 (0.65 - 1.04)	0.83 (0.70 - 0.98)*
**Knowledgeable of ANC services**				
≤ 2 services	57(35.2)	162	1	1
≥3 services	158(95.8)	165	2.72 (2.20 - 3.36)***	2.77 (2.24-3.42)***
**Intends spouse to carry another pregnancy**				
No	35 (76.1)	46	1	
Yes	177 (63.7)	276	0.83 (0.69 - 1.00)	0.83 (0.71-0.97)*
**Health information mainly obtained from health facility worker**				
No	115(54.0)	189	1	
Yes	98(46.0)	138	1.16 (0.99 - 1.36)	1.14 (1.01 - 1.29)*
**Last delivery of spouse was at health facility**				
No	28 (48.3)	58	1	
Yes	187 (69.0)	271	1.42 (1.08 - 1.88)*	1.31 (1.04-1.64)*

log likelihood = - 270.43

*p < 0.05, **p < 0.01 ***p < 0.001

## Discussion

In this study, the level of male attendance of skilled ANC was relatively high at 65.4% (60.3 - 70.5) as compared to 42% for Omoro county in Gulu observed from district health management information system (HMIS) reports in 2008 [22] and much higher than the 4% national average [20]. This study reports relatively high levels of male attendance of ANC possibly because the sample was composed of peri-urban males who arguably have better access to health facilities where integrated ANC/PMTCT services are provided than the rural that make up the majority of the population. Studies in Sub-Saharan localities where PMTCT programmes are being implemented as part of the ANC services report male attendance rates at 12.5% in northern Tanzania [26], about 15 and 16% respectively at different clinics in Nairobi, Kenya [15,27] and 25% in Côte d'Ivoire[19].

HIV screening was perceived as the main benefit to ANC attendance. Although it was not significantly associated with male partner ANC attendance, it is of public health importance because HIV screening is one of the quality services offered at ANC [2,20]. Furthermore HIV testing is meant to benefit the couple so that assessment of the risk of HIV infection to the unborn child is done during pregnancy and prevented. In case of couples that are HIV discordant, male attendance presents the opportunity for health providers to promote use of barrier contraceptive methods such as condoms. The perceived benefit to male partners of monitoring foetal growth is similarly of public health importance, even though it was not found significantly associated with male attendance of ANC. A study conducted in Taiwan reported anxiety of the male partner about the newborn during the wife's pregnancy to increase their participation in maternal health care [28]. Perceived benefit of foetal monitoring during spousal pregnancy is worth inclusion in subsequent studies to determine whether it is a predictor of male attendance of maternal health care.

The main factors identified by the respondents in the survey as barriers to attendance of ANC were lack of transportation, long waiting time and long walking distance to a health facility. Perceiving the walking distance to the health facility as long (greater than one-hour walk or greater than 5 km) was a prominent barrier and associated with reduced male attendance of ANC (p = 0.034). Long distance as a barrier to male attendance of maternal health care has been reported by other cross-sectional studies in Tanzania, Uganda and Bangladesh [29-31]. This finding emphasizes the need for the health sector to further increase access to maternal health services.

On the other hand, males that wanted their spouses to carry another pregnancy were found to be associated with reduced attendance of ANC. This finding could suggest that fertility preferences are modified once a man attends ANC with the spouse during pregnancy. This is possible because health education about family planning which is provided during the pre and postnatal visits encourages child spacing for at least two years[8]. We however also found that male partners with high fertility preferences were less likely to have four or more children, implying that they wanted to have more children because they perceived the number they had as few. Given that total fertility rate in Uganda is 6.7% [5,32], the latter may be the alternative explanation for this finding.

Interestingly, we found that male attendance of ANC was associated with higher likelihood of spouse having skilled delivery at her last childbirth. To the best of our knowledge, this is a new finding; however, some community-level intervention studies found health education and support programmes to increase skilled attendance at childbirth [9-12].

Knowledge about safe motherhood services among the male partners was limited. Only 47.1% respondents knew 3 - 5 services offered at ANC. This finding is consistent with other studies in Taiwan, India and Tanzania where male partners were found to have limited knowledge about safe motherhood and sexuality [28,33-36]. In Uganda, this finding is not surprising because male attendance of safe motherhood is a new approach that was introduced with the national PMTCT program in 2001 [20]. Limited knowledge of male partners about maternal health issues has been reported to be a significant barrier to their attendance [34,37]. In this study, male partners that had reasonable knowledge of services offered at ANC (at least three services) were significantly more likely to attend ANC (p < 0.001). This finding supports the evidence that increasing male partner's knowledge of safe motherhood is positively associated with attendance of ANC [38]. However, the study design does not allow us to conclusively affirm the temporal relation between knowledge and ANC attendance. It is also possible that the more one attends ANC, the more knowledgeable one becomes about ANC services.

Obtaining health information from a facility health worker was positively associated with male partner attendance of ANC with their spouses. The finding suggests that males regard health workers as a credible source of health information, and this is influential on their health behaviour. This finding supports evidence from a systematic review by Sternberg et al (2004) that concluded; health promotion for male involvement in sexual and reproductive health programs has a high promise of success [34]. The alternative explanation to this finding is that male attendance of ANC was positively associated with obtaining health information from facility health workers, which would point towards effectiveness of health education at ANC[38].

We found that male partner's education was not significantly associated with attendance of ANC in the adjusted analysis. The interpretation of this result is that the variable is collinear with knowledge of ANC services, and we therefore omit it from the final model. A cross-sectional study conducted in Nigeria [39] that interviewed pregnant women, who are considered surrogate respondents in this comparison, showed that low education was associated with reduced utilization of ANC similar to other studies [30,31].

## Study Limitations

Recall bias could have been present even after restricting inclusion to males whose spouses last delivered 24 months prior to the survey. Two years is a long period, yet pregnancy may not signify as much importance to the male partner as it does to the female. Additionally reporting bias arising from men wanting to provide socially desirable responses especially regarding the level of ANC attendance could have been a potential pitfall to the study. This could have been overcome by cross validation of the men's responses by women interviews.

In this study, we used multi-stage cluster sampling but analyzed subjects at individual level, which could have potentially reduced the statistical efficiency at analysis[40]. We used a conservative design effect of 1.5 to cater for the sampling error, but this may not have been sufficient[40].

Our study population was largely peri-urban, therefore our findings may not be entirely generalizable to the rest of Gulu district and other rural settings in Uganda. However, we gain insights into how similar populations can be targeted to improve male ANC attendance.

This study design was cross-sectional which limits us from making any causal inferences in relation to the main outcome and independent variables.

## Conclusions

Male attendance of skilled ANC of a peri-urban population in Gulu was high at 65.4% compared to 42% in the rest of the district and an estimated 10% attendance rate in the other regions in Uganda. Willingness to attend subsequent skilled ANC was nearly universal. Male partners had favourable perceptions for attendance of skilled ANC that included foetal growth monitoring, HIV screening and identifying complications during pregnancy.

Factors associated with male attendance of ANC were: knowledge of at least three ANC services, obtaining health information from facility health workers, wife last gave birth at a health facility, distance to nearest health facility less than 1 hour or 5 km and the male partner having no intention for the spouse to carry another pregnancy.

Knowledge about ANC services was the main independent variable associated with male attendance of ANC with their spouses. We recommend that governments and local health authorities should develop strategies to empower men (both in communities and at health facilities) with knowledge about ANC. Such strategies may contribute to increasing male attendance in ANC, improve skilled attendance at delivery and improve maternal health outcomes. Strategies that increase male attendance of skilled ANC are likely to be feasible in post-conflict regions and resource limited settings.

## Competing interests

The authors declare that they have no competing interests.

## Authors' contributions

RT: Designed the study, coordinated recruitment of participants, collected and entered data, analyzed data and drafted the manuscript. JNS: Designed the study, participated in data analysis and reviewed all drafts of the manuscript. JKL: Designed the study, participated in data analysis, reviewed all drafts of the manuscript. NTM: Guided data analysis, reviewed all drafts of the manuscript. All authors read and approved the final manuscript.

## Pre-publication history

The pre-publication history for this paper can be accessed here:

http://www.biomedcentral.com/1471-2393/10/53/prepub
